# Effect of electroacupuncture on glucose and lipid metabolism in type 2 diabetes: A protocol for systematic review and meta-analysis

**DOI:** 10.1097/MD.0000000000027762

**Published:** 2021-12-10

**Authors:** Yiran Han, Zeyuan Lu, Shaotao Chen, Tianjiao Gao, Xiaochao Gang, Ting Pan, Meng Meng, Mingjun Liu

**Affiliations:** a Department of Acupuncture and Tuina, Changchun University of Chinese Medicine, Changchun, China; b Department of Pharmacy, The Second Hospital of Jilin University, Jilin, China; c Acupuncture and Massage Center of the Third Affiliated Clinical Hospital of Changchun University of Chinese Medicine, Changchun, China.

**Keywords:** electroacupuncture therapy, meta-analysis, protocol, type 2 diabetes mellitus (T2DM)

## Abstract

**Background:**

Type 2 diabetes mellitus (T2DM) is a global pandemic with a significant negative impact on health-related quality of life. Worldwide, the prevalence of T2DM has almost doubled since 1980. Although multiple systematic reviews and clinical trials have suggested that electroacupuncture could be effective for T2DM treatment, whether it can improve glucose and lipid metabolism has not been systematically reviewed.

**Methods:**

We searched PubMed, Medline, Embase, Web of Science, Cochrane Central Register of Controlled Trials, China National Knowledge Infrastructure, Chinese Biomedical Literature Database, Chinese Scientific Journal Database, and Wan-Fang Database from the date of creation to December 2022. Language is limited to both Chinese and English languages. Clinical randomized controlled trials related to acupuncture for T2DM were included in this study. Fasting plasma glucose, fasting insulin, lipid profile, and glycated hemoglobin levels were the primary outcomes. In addition, we manually retrieved other resources, including reference lists of identified publications, conference articles, and gray literature. Research selection, data extraction, and research quality assessments were independently completed by 2 researchers.

**Results:**

This study provides more options for clinicians and patients to treat obese patients with type 2 diabetes.

**Conclusion:**

In this study, we aimed to summarize and assess the effectiveness and safety of EA as a supplemental method to treat T2DM patients from clinical trials and provide more options for clinicians and patients to treat T2DM.

**Trial registration:**

This study was registered at INPLASY with registration number INPLASY202180008 (https://inplasy.com/inplasy-2021-8-0008/).

**Systematic review registration:**

INPLASY202180008.

## 1 Introduction

Type 2 diabetes mellitus (T2DM) is characterized by hyperglycemia, a relative lack of insulin and insulin resistance, and is a chronic metabolic disease. The incidence rate of T2DM is increasing dramatically worldwide. The accompanying hyperglycemic state will cause abnormal metabolism of the patients’ metabolism, not only cause diabetic nephropathy and other related complications, but also increase the coronary atherosclerosis significantly, and the incidence of coronary atherosclerosis is increased significantly in 2 cases. The prevalence of cardiovascular and cerebrovascular diseases such as myocardial infarction.^[1]^ With the aging of the global population and changes in lifestyle, T2DM has developed from a rare disease to a common disease worldwide. In China alone, the prevalence of diabetes has increased tenfold in 30 years, from 0.67% in 1980 to 10.4% in 2013.^[2]^ From 2015 to 2017, the epidemiology of thyroid, iodine nutritional status, and diabetes mellitus in 31 provinces of China conducted by the endocrinology branch of the Chinese Medical Association showed that the prevalence of diabetes in people aged 18 and above was 11.2%.^[3]^ With the development of science and technology, people's understanding of T2DM has gradually deepened, and the detection and treatment methods for T2DM are gradually increasing. In addition to a variety of therapeutic drugs and metabolic surgery for obese T2DM patients, traditional Chinese medicine has also gradually played a role in the treatment of T2DM.

Acupuncture, the most distinctive external therapy in traditional Chinese medicine, has long been applied to assist in the control of blood glucose and lipid levels in T2DM patients. Electroacupuncture is a perfect combination of modern science, technology, and acupuncture therapy. Animal experimental studies have shown that electroacupuncture at different frequencies can activate cholinergic nerves and stimulate the release of β-endorphin and other endogenous opioid peptides by the adrenal glands, thus promoting the secretion of insulin.^[4–6]^

Although multiple systematic reviews and clinical trials have suggested that electroacupuncture could be effective for T2DM treatment, whether it can improve glucose and lipid metabolism has not been systematically reviewed. This meta-analysis aimed to summarize and assess the effectiveness and safety of EA as a supplemental method to treatT2DM patients from clinical trials and provide more options for clinicians and patients to treat T2DM.

## 2 Methods and analysis

The systematic review was performed following the guidelines of the Preferred Reporting Items for Preferred Reporting Items for Systematic Review and Meta-Analysis Protocols 2015.^[7]^ This protocol was registered on the International Platform of Registered Systematic Review and Meta-analysis Protocols (INPLASY202180008).

### 2.1 Inclusion criteria

#### 2.1.1 Types of studies

The type of literature gathered must be based on random clinical research (RCT) trials. Non-RCTs will be ruled out, including studies such as case reports and studies without sufficient information about the randomized method or process. Do not limit the use of blind methods, but the authors should clearly claim that they performed random grouping.

#### 2.1.2 Types of participants

The diagnosis of T2DM was based on the diagnostic criteria for diabetes in 1999. Meanwhile, the participants who were included had no limitations in age, sex, region, citizenship, and nationality. Cases related to serious diseases, pregnancy, and drug-induced T2DM were excluded.

#### 2.1.3 Types of interventions and comparisons

One therapeutic intervention used in the experimental group included EA. Therefore, acupoint catgut embedding, cupping, acupoint injection, blood-letting therapy, and herbal medicine should be excluded.

#### 2.1.4 Types of outcome measures

The primary outcome measures will be fasting plasma glucose, glycated hemoglobin, fasting insulin, and lipid profile. In addition. Weight loss (kg), homeostatic model assessment of insulin resistance level, C-reactive protein level, dropout, side effects, and rebound will be assessed.

### 2.2 Data source

We electronically searched PubMed, Embase, Web of Science, the Cochrane Central Register of Controlled Trials, China National Knowledge Infrastructure, Chinese Biomedical Literature Database, Chinese Scientific Journal Database, and Wan-Fang Database from the date of creation to December 2022. In addition, we manually retrieved other resources, including reference lists of identified publications, conference articles, and gray literature.

### 2.3 Search strategy

We constructed a search strategy using a combination of medical subject heading terms and text words. We will verify the systematic review and meta-analysis that also focused on our topic and the bibliographies of eligible studies in order to add any additional eligible studies. For example, we summarized the search strategy for PubMed (Table 1).

**Table 1:** Search strategy used in PubMed.

NO.	Search terms
#1	Type 2 diabetes mellitus (All files)
#2	Type 2 diabetes (All files)
#3	T2DM (All files)
#4	#1 OR #2 OR #3
#5	Electroacupuncture (All files)
#6	Electro-acupuncture (All files)
#7	Complementary therapy of electroacupuncture (All files)
#8	#5 OR #6 OR #7
#9	Randomized controlled trial (All files)
#10	Controlled clinical trial (All files)
#11	Randomly (All files)
#12	Randomized (All files)
#13	Placebo (All files)
#14	Double-blind method (All files)
#15	Single blind method (All files)
#16	Trials (All files)
#17	#9 OR #10-16
#18	#4 AND #8 AND #17

### 2.4 Studies selection

**Figure 1:**
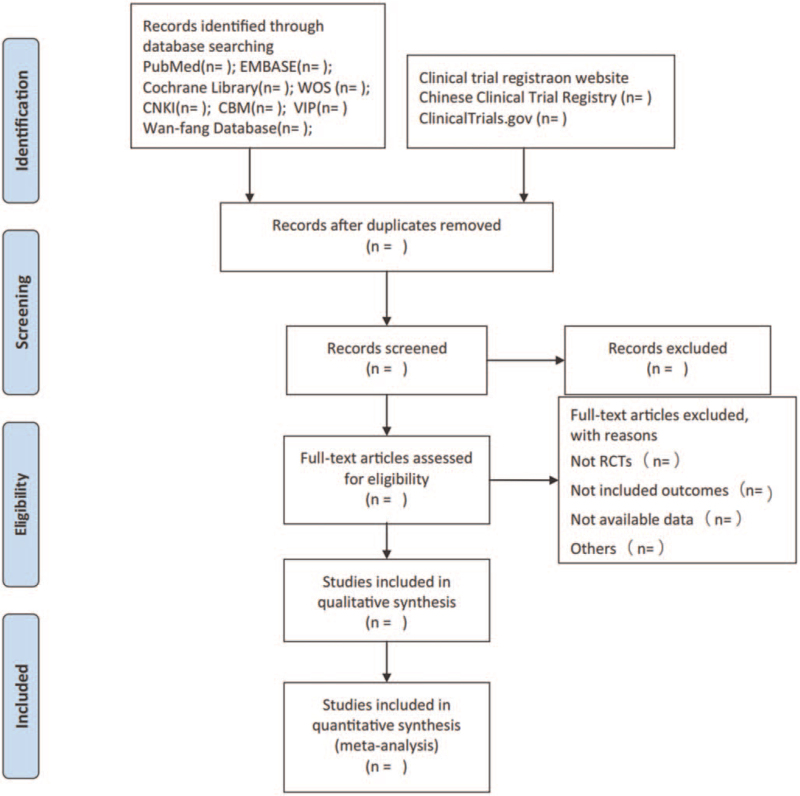
Flow diagram of study selection process.

Studies will be identified using NoteExpress 3.2.0. After the initial removal of duplicate studies, 2 reviewers independently screened the titles and abstracts based on the eligibility criteria. Full-text studies will be retrieved for all potentially includable SR or SR protocols. If studies contain insufficient information to make a decision about eligibility, they will try to contact authors of the original reports to obtain further details. The process of identification and selection of literature is shown in Figure 1. During the procedure, any disagreement about the retrieval of eligible studies will be settled by consulting a third senior author.

### 2.5 Statistical analysis

We will use Review Manager 5.4 software provided by the Cochrane Collaborative Network for statistical analysis. For continuous variables, the mean and SD of each study were obtained and pooled as the mean difference or standardized mean difference with a 95% confidence interval. Statistical heterogeneity analysis was performed for the included RCTs. The Cochrane *I*^2^ test was used for the statistical analyses. When *I*^2^ was <50% or *P* > .05, there was no statistical heterogeneity between the studies, and the fixed-effect model was selected to combine the effect amount; otherwise, the random-effects model was adopted.

### 2.6 Assessment of heterogeneity

Statistical heterogeneity among the studies was assessed using the *Q* test and *I*^2^ statistics. According to the Cochrane Handbook, the thresholds for the interpretation of the *I*^2^ statistics are as follows: 0% to 40%, might not be important; 30% to 60%, may reflect moderate heterogeneity; 50% to 90%, may indicate substantial heterogeneity; 75% to 100%, represents considerable heterogeneity^[8]^ After statistical heterogeneity is established, the researchers will search for possible sources from the clinical and methodological perspectives and perform subgroup or sensitivity analysis to detect the possible causes of heterogeneity.

### 2.7 Assessment of publication bias

We used funnel charts to assess reporting biases. When a sufficient number of included studies (at least 10 trials) are available, we will conduct a test for funnel plot asymmetry using the Egger method.

### 2.8 Sensitivity analysis

To test the robustness of the main decisions in the review process, we conducted a sensitivity analysis. The main analysis points included the impact of method quality, sample size, and missing data on the study. The meta-analysis will be reused, and more inferior-quality studies will be excluded. The results are compared and discussed based on the results.

### 2.9 Grading the quality of evidence

The quality of systematic reviews will be evaluated using the grading of recommendations, assessment, development, and evaluation. Five downgrading factors, including risk of bias, inconsistency, indirectness, imprecision, and publication bias, were assessed. The assessment results were divided into 4 levels: high, moderate, low, or very low.

## 3 Discussion

In recent years, with the continuous accumulation of evidence-based medical evidence for T2DM and the change of disease spectrum, people's understanding of T2DM has gradually changed.^[9]^ With the arrival of the aging era, T2DM has posed a serious burden to many families and society.^[10]^ As a metabolic disease characterized by hyperglycemia, with the continuous elevation of blood glucose, the body will develop from normal to prediabetes, and then develop into diabetes and complications. In this process, any intervention measures such as improving life, diet, and exercise habits can improve the pathological state leading to the increase of blood glucose^[11–13]^ (such as impaired insulin secretion, insulin resistance, obesity, etc), help the body slow down the increase of blood glucose, and even reverse the hyperglycemia to a lower level.

However, the current treatment for T2DM can only be hypoglycemic drugs for the direct cause, but long-term drug use has also influenced people's lives and work. In addition, many complications caused by poorly controlled diabetes have a significant impact on quality of life. Meanwhile, more and more patients began to realize the single limitation of controlling diseases only by hypoglycemic drugs, so they sought the help of traditional Chinese medicine from the hospital of traditional Chinese medicine. Therefore, how to use traditional Chinese medicine to prevent and improve the symptoms of T2DM interventions and find an intervention method that can improve insulin resistance and reduce side effects in patients with high glucose and high fat T2DM has become the main research direction of T2DM, and is also one of the focuses of traditional Chinese medicine in the field of diabetes research. In this study, we aimed to summarize and assess the effectiveness and safety of EA as a supplemental method to treat T2DM patients from clinical trials and provide more options for clinicians and patients to treat T2DM.

## Author contributions

**Conceptualization:** Yiran Han, Xiaochao Gang.

**Data curation:** Yiran Han, Zeyuan Lu.

**Formal analysis:** Shaotao Chen, Meng Meng.

**Methodology:** Zeyuan Lu, Ting Pan.

**Software:** Yiran Han, Tianjiao Gao.

**Supervision:** Mingjun Liu.

**Writing – original draft:** Yiran Han, Xiaochao Gang.

**Writing – review & editing:** Zeyuan Lu, Mingjun Liu.

## References

[1] Skyler JS. Diabetes mellitus: pathogenesis and treatment strategies. J Med Chem 2004;47:4113–7. 15293979 10.1021/jm0306273

[2] Endocrinology CSO, Society CD Association CE, et al. Expert consensus on China's pre diabetes intervention [J]. Chin J Endocrine Metab, 2020, 371-380.

[3] Society CD. Guideline for prevention and treatment of type 2 diabetes in China (2020 Edition). Chin J Diabetes 2021;13:315–409.

[4] Chang SL Lin JG Chi TC. An insulin-dependent hypoglycaemia induced by electroacupuncture at the Zhongwan (CV12) acupoint in diabetic rats. Diabetologia 1999;42:250–5. 10064107 10.1007/s001250051146

[5] Lin JG Chang SL Cheng JT. Release of beta-endorphin from adrenal gland to lower plasma glucose by the electroacupuncture at Zhongwan acupoint in rats. Neuroscience Letters 2002;326:17–20. 12052528 10.1016/s0304-3940(02)00331-2

[6] Chung Y-C Chen Y-I Lin C-M. Electroacupuncture combined with acarbose improves insulin sensitivity via peroxisome proliferator-activated receptor γ activation and produces a stronger glucose-lowering effect than acarbose alone in a rat model of steroid-induced insulin resistance. Acupunct Med 2020;38:335–42. 32297559 10.1177/0964528419901135

[7] Shamseer L Moher D Clarke M. Preferred reporting items for systematic review and meta-analysis protocols (PRISMA-P) 2015: elaboration and explanation. BMJ Clin Res 2015;349: 10.1136/bmj.g7647 25555855

[8] Higgins JPT, Green S (editors). Cochrane Handbook for Systematic Reviews of Interventions Version 5.0.1 [updated September 2008]. The Cochrane Collaboration, 2008. Available from: www.cochrane-handbook.org.

[9] Dajin Z Zheng Z Linong J. China expert consensus on alleviating type 2 diabetes mellitus. Chin Gen Med 2021;24:4037–48.

[10] Zheng Y Ley SH Hu FB. Global aetiology and epidemiology of type 2 diabetes mellitus and its complications. Nat Rev Endocrinol 2018;14:88–98. 29219149 10.1038/nrendo.2017.151

[11] Gong Q Zhang P Wang J. Morbidity and mortality after lifestyle intervention for people with impaired glucose tolerance: 30-year results of the Da Qing Diabetes Prevention Outcome Study. Lancet Diabetes Endocrinol 2019;7:452–61. 31036503 10.1016/S2213-8587(19)30093-2 PMC8172050

[12] Pan XR Li GW Hu YH. Effects of diet and exercise in preventing NIDDM in people with impaired glucose tolerance. The Da Qing IGT and Diabetes Study. Diabetes Care 1997;20:537–44. 9096977 10.2337/diacare.20.4.537

[13] Knowler WC Barrett-Connor E Fowler SE. Reduction in the incidence of type 2 diabetes with lifestyle intervention or metformin. N Engl J Med 2002;346:393–403. 11832527 10.1056/NEJMoa012512 PMC1370926

